# The Epitranscriptomic Landscape of Gastric Cancer Stem Cells: The Emerging Role of m^6^A RNA Modifications

**DOI:** 10.3390/cancers17213589

**Published:** 2025-11-06

**Authors:** Diana Pádua, Patrícia Mesquita, Raquel Almeida

**Affiliations:** 1 i3S—Institute for Research and Innovation in Health, University of Porto, 4200-135 Porto, Portugal; dspadua@icbas.up.pt (D.P.); ralmeida@ipatimup.pt (R.A.); 2 IPATIMUP—Institute of Molecular Pathology and Immunology, University of Porto, 4200-465 Porto, Portugal; 3 ICBAS—School of Medicine and Biomedical Sciences, University of Porto, 4050-313 Porto, Portugal; 4 Biology Department, Faculty of Sciences, University of Porto, 4169-007 Porto, Portugal

**Keywords:** gastric cancer, cancer stem cells, N^6^-methyladenosine (m^6^A), epitranscriptomics, biomarkers, RNA modifications, therapy resistance

## Abstract

Cancer stem cells (CSCs) represent a small but critical subpopulation of tumor cells that drive therapy resistance, relapse and metastasis. Gastric cancer stem cells (GCSCs) have been identified through surface markers and transcriptional signatures, revealing their central role in tumor progression. Recently, N^6^-methyladenosine (m^6^A) RNA modification has emerged as a crucial epitranscriptomic regulator of CSC biology. The m^6^A machinery, including “writers” (METTL3, METTL14, WTAP, VIRMA), “erasers” (FTO, ALKBH5) and “readers” (YTHDFs/ YTHDCs, IGF2BPs, hnRNPA2B1), orchestrates RNA stability, splicing, translation and decay, thereby influencing self-renewal and oncogenic signaling. In GCSCs, m^6^A controls pluripotency factors, oncogenic transcripts and non-coding RNAs, collectively reinforcing stemness and malignant potential. Mounting evidence implicates dysregulated m^6^A effectors as not only key drivers of GCSC biology but also as promising biomarkers for patient stratification and therapeutic targets capable of selectively eliminating CSCs. Harnessing this knowledge could enable earlier diagnosis, more accurate patient stratification and more precise treatments. However, challenges remain regarding the resolution of m^6^A profiling, therapeutic selectivity to avoid unwanted toxicity and biomarker validation for clinical use. This review summarizes the discovery and features of CSCs, highlights the functional role of m^6^A in GCSCs, and explores diagnostic and therapeutic opportunities while outlining key difficulties for clinical translation.

## Simple Summary

Why does gastric cancer frequently recur despite aggressive treatment? The answer may lie in a small but stubborn population of cells hidden deep within the tumor, the cancer stem cells (CSCs). These cells possess self-renewal capacity, resist conventional therapies and drive relapse and metastasis, making them critical determinants of patient outcomes. Their persistence highlights the urgent need to understand the molecular mechanisms sustaining their survival. One emerging explanation comes from the field of epitranscriptomics, which investigates chemical modifications of RNA that regulate gene expression post-transcriptionally. Among these modifications, N^6^-methyladenosine (m^6^A) has attracted particular attention. It serves as a dynamic regulator of RNA metabolism, controlling stability, translation and splicing, and has been shown to promote stemness and aggressiveness in gastric cancer stem cells (GCSCs). Dissecting the m^6^A-dependent regulatory networks in CSCs paves the way for novel diagnostic biomarkers and therapeutic strategies designed to eliminate the root of gastric cancer recurrence and progression.

## 1. Introduction

Gastric cancer remains one of the most serious health challenges, ranking among the top five most common and deadly cancers in the world [1]. Despite advances, both in early detection tools and therapeutic strategies, patient prognosis remains poor [2]. Additionally, no two gastric cancers are the same, reflecting the disease’s heterogeneity across distinct molecular subtypes and variations within the tumor microenvironment, which complicates diagnosis and treatment [3]. Within this complexity, cancer stem cells (CSCs) have emerged as key drivers of tumor initiation, progression, metastasis and relapse even after a seemingly successful treatment [4]. Gastric cancer stem cells (GCSCs) have shown to be resistant to conventional therapies and capable of renewing tumors, making them a critical therapeutic target. Several surface markers, including CD44, ALDH1, LGR5 and CD133, along with transcription factors such as SOX2 and HMGA1, are commonly used to identify and isolate GCSCs [5,6,7,8]. Understanding the molecular vulnerabilities of these cells, as well as the regulatory networks that sustain their stem-like properties, is essential for the development of precise diagnostic tools and personalized biomarker-driven therapies that can improve patient outcomes [5,6,9]. In recent years, a new layer of cancer biology has captured attention—RNA modifications—adding a new dimension to our understanding of cancer biology [10,11]. Among these, N^6^-methyladenosine (m^6^A) has emerged as the most prevalent and functionally significant internal mRNA modification in eukaryotic cells [11]. The m^6^A landscape is dynamically regulated by three classes of proteins: “writers” (e.g., METTL3, METTL14, WTAP and VIRMA), “erasers” (e.g., FTO and ALKBH5) and “readers” (e.g., YTHDF, YTHDC, IGF2BP family members and hnRNPA2B1) [12,13]. These factors collectively control key aspects of RNA metabolism, including splicing, export, stability and translation, influencing cell differentiation, development and disease progression [14]. Dysregulation of the m^6^A machinery has been increasingly linked to tumor growth, therapy resistance and the maintenance of stem-like traits in various cancers [15,16]. While m^6^A biology has been explored extensively in other tumor types, its role in GCSCs is only beginning to be unraveled. Aberrant expression of m^6^A-related enzymes has already been associated with aggressive tumor behavior, poor prognosis and treatment resistance in gastric cancer [17]. However, the precise molecular mechanisms by which m^6^A influences GCSC biology remain incompletely understood. This review aims to bring together the current knowledge on the intersection between m^6^A RNA methylation and GCSCs, highlighting how this emerging epitranscriptomic layer shapes stemness and tumor behavior. We also explore the therapeutic potential of targeting m^6^A regulators as a novel strategy to eradicate GCSCs and ultimately improve treatment outcomes for gastric cancer patients.

## 2. Gastric Cancer Stem Cells Overview

In the past three decades, CSCs have attracted significant attention due to their role in tumor biology. In 1964, Kleinsmith and Pierce demonstrated that a single mouse embryonal carcinoma cell, derived from a teratocarcinoma, was capable of regenerating the complete histopathological features of the original tumor [18]. Notably, embryonal carcinoma cells represent a small stem cell–like subpopulation within teratocarcinomas. These defining properties—tumor regeneration from a single cell and the presence of a self-renewing subpopulation—constitute the fundamental hallmarks of CSCs. The first evidence for the existence of human CSCs came in 1994, when Lapidot and colleagues identified a rare CD34+/CD38− cell subpopulation in acute myeloid leukemia capable of initiating disease in immunodeficient mice [19]. A few years later, Bonnet and Dick confirmed that these cells alone could reproduce the disease, establishing the concept that only a fraction of cancer cells possess the potential to initiate the tumorigenic process [20]. This discovery was soon extended to solid tumors, where it was validated that only specific subsets of cells were able to initiate tumors in vivo [21,22]. Since then, CSCs have been described in a wide range of cancers and are now recognized as key contributors to tumor development, therapy resistance, metastasis and relapse [23]. Biologically, CSCs are defined by their ability to self-renew, to differentiate into diverse tumor cell lineages (asymmetric division) and to adapt under stressful conditions such as chemotherapy, radiotherapy or hypoxia [24]. They are usually identified by a combination of functional assays and cell-surface markers, which vary according to tumor type. For example, in breast cancer, CD44+/CD24+ enriched cells displayed cancer stem-like features while CD133 is frequently used in colon and brain tumors and CD90 in liver tumors [25,26,27,28]. Importantly, the transplantation of only a few hundred CSCs into immunodeficient mice can be sufficient to regenerate an entire tumor, illustrating their remarkable tumorigenic potential [24].

**Figure 1:**
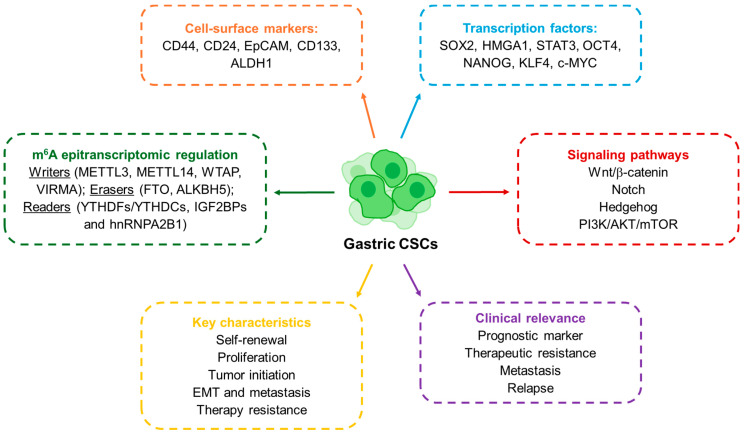
Schematic representation of the molecular and functional features of GCSCs.

GCSCs share these core CSC properties but also exhibit features unique to gastric cancer biology. They were first characterized in 2007, when Takaishi and colleagues reported that CD44+ cells exhibited spheroid-forming ability and could initiate tumors in vivo, fulfilling the criteria of cancer stem-like cells [29]. Since then, several markers have been proposed to define gastric GCSCs (Figure 1). CD44 remains the most widely studied, but other molecules such as CD24, EpCAM, CD54, CD90, CD133, CXCR4, Lgr5 and ALDH1 have also been associated with GCSC populations [6,9,30]. In parallel, transcription factors central to pluripotency such as SOX2, OCT4 and NANOG have been shown to regulate GCSCs characteristics, while signaling pathways like WNT, NOTCH, Hedgehog, NF-κB and TGF-β/SMAD provide the molecular framework that sustains their activity [6,31,32]. Thus, these molecules are also key to identifying and studying GCSCs. For instance, using the SORE6-GFP reporter system, engineered to detect transcriptional activity of SOX2 and OCT4, we were able to identify and isolate GCSCs (SORE6+ cells). These cells exhibited enhanced stemness characteristics, including increased self-renewal capacity, elevated tumor-initiating potential and resistance to conventional chemotherapeutic agents such as 5-fluorouracil (5-FU) [7]. Clinically, the expression of CSC-associated markers is linked to more aggressive disease and worse outcomes: for instance, CD44 has been validated as an independent prognostic factor, while high levels of OCT4 or SOX2 correlate with metastasis and poor survival [33,34,35,36]. A more recent layer of complexity has been added by the discovery of the role of RNA modifications in CSC regulation. Among them, N^6^-methyladenosine (m^6^A) has emerged as the most abundant internal modification in eukaryotic mRNA, regulating RNA metabolism, including stability, splicing and translation [14]. The deposition and interpretation of m^6^A marks depend on a set of enzymes commonly referred to as “writers” (METTL3, METTL14, WTAP), “erasers” (FTO, ALKBH5), and “readers” (YTHDF, IGF2BP and hnRNPA2B1 proteins) [37]. Studies in several cancers show that m^6^A modifications are central for the maintenance of stem-like features and therapy resistance in CSCs [38,39]. In gastric cancer, the relevance of m^6^A regulation is becoming increasingly clear. Elevated expression of METTL3 or WTAP, as well as decreased levels of METTL14, have been associated with poor patient prognosis [40,41]. More strikingly, recent work has shown that m^6^A modification of long non-coding RNAs in GCSCs can promote stemness and tumorigenicity by stabilizing transcripts and enhancing proliferative pathways [41]. Taken together, the evidence underscores how the CSC concept has evolved: from its first description in leukemia, to its establishment in solid tumors, and more recently to its epitranscriptomic regulation. In gastric cancer, the identification of CSCs through surface markers and transcriptional networks has already provided important insights into disease aggressiveness and therapeutic resistance. The emerging role of m^6^A adds a powerful new dimension, suggesting that RNA modifications could serve not only as biomarkers for GCSCs but also as actionable targets in the development of future therapies.

## 3. The m^6^A Modification Machinery in the Gastric Cancer Stem Cell Phenotype

Among the regulatory layers that shape CSC biology, RNA modifications, prominently m^6^A, have recently come into the spotlight. In gastric cancer, increasing evidence indicates that m^6^A modifications not only correlate with tumor progression but also play a role in maintaining the malignant phenotype of GCSCs [42]. The enzymes that establish, remove, and interpret this mark, often referred to as the m^6^A machinery, form a dynamic system that can profoundly influence cell fate decisions. An overview of their roles is provided below and in Table 1.

### 3.1. “Writers”

The “writers” of m^6^A marks are methyltransferase complexes that deposit the modification on specific adenosine residues. Central to this activity are METTL3 and METTL14 methyltransferases, stabilized by the cofactor WTAP, along with accessory proteins such as RBM15, KIAA1429 (VIRMA) and ZC3H13, which confer substrate specificity [96,97,98]. These enzymes are responsible for the global landscape of m^6^A in cells, and their dysregulation has been linked to stemness, tumor initiation, metastasis and resistance to therapy in multiple cancers. Overall, a consistent pattern emerges, METTL3 generally functions as an oncogenic driver that enhances stem-like properties through m^6^A-dependent stabilization of key transcripts. In gastric cancer, METTL3-driven m^6^A methylation at specific adenosines on the long non-coding RNAs (lncRNAs) PSMA3-AS1 (A1225) and MIR22HG (A2041) is enriched in GCSCs compared with non-stem cells [41]. Silencing METTL3 reduced GCSC viability, induced G_0_/G_1_ arrest, and promoted apoptosis, highlighting the role of m^6^A in stemness maintenance. Remarkably, site-specific methylation rescue restored proliferative and tumorigenic potential by stabilizing EEF1A1 and LRPPRC, thereby suppressing apoptosis and reestablishing self-renewal in vivo [41,43]. These findings identify site-specific m^6^A methylation of lncRNAs as a molecular switch sustaining GCSC survival and malignant progression [41]. Moreover, studies with oxaliplatin sensitive and resistant gastric cancer organoids revealed that CD133^+^ CSCs acquire resistance through METTL3-mediated m^6^A modification of PARP1 mRNA. This modification stabilizes PARP1, enhances DNA repair, and promotes oxaliplatin resistance, advancing our understanding of drug resistance mechanisms in gastric cancer [44]. Evidence from other tumors supports the notion that METTL3 often acts as an oncogenic driver of CSC phenotypes. For example, in colorectal CSCs, METTL3 promotes WNT/β-catenin signaling, sustaining stemness [99]. In glioblastoma CSCs, METTL3-mediated m^6^A methylation enhances SOX2 expression, reinforcing self-renewal and tumorigenic capacity [39]. In leukemia, overexpression of METTL3 has been shown to increase MYC pathway activity, supporting leukemic CSCs survival [100]. These studies highlight METTL3 as a master regulator that reinforces CSC phenotypes through transcript stabilization and pathway activation. In contrast, METTL14 exhibits context-dependent and often opposing roles. In gastric cancer, METTL14 suppresses stemness, and low METTL14 expression correlates with poor survival. Mechanistically, it promotes m^6^A-dependent degradation of ATF5 mRNA and restrain the WDR74/β-catenin axis [48]. Also, loss of METTL14 suppresses differentiation and promotes stem-like traits in acute myeloid leukemia [101]. This points to a nuanced role of “writer” components depending on tumor type and cellular context. Finally, in what concerns WTAP, it stabilizes the METTL3/METTL14 complex, and its overexpression in gastric cancer predicts poor prognosis and therapy resistance [50,51].

Taken together, these studies reveal that the interplay between METTL3, METTL14 and WTAP fine-tunes m^6^A methylation activity, influencing the balance between CSC maintenance and differentiation across tumor types.

### 3.2. “Erasers”

Counterbalancing the “writers” are the “erasers”, enzymes that remove m^6^A and thereby restore transcripts to an unmethylated state. The first to be identified, fat mass-and obesity-associated protein (FTO), and later AlkB homolog 5 (ALKBH5), provide CSCs with plasticity, allowing dynamic remodeling of the transcriptome in response to stress, therapy or microenvironmental cues [102,103]. Recent studies showed that FTO promotes stem-like properties and lymph node metastasis in gastric cancer, while its knockdown suppresses proliferation, migration, and invasion [55,56]. Mechanistically, FOXA2 suppresses FTO transcription, while FTO promotes stemness by stabilizing MYC mRNA and acting through SOX2 [55,57]. ALKBH5 plays a context-dependent role in gastric cancer, reported as either upregulated or downregulated across studies [61,64,65]. Functionally, it promotes cisplatin resistance via the HIF-1α pathway and enhances N-Methyl-N’-nitro-N-nitrosoguanidine (MNNG)-induced stemness and invasion through activation of the ZKSCAN3–VEGFA axis [61]. In addition, lncNRON facilitates ALKBH5 recognition of m^6^A-modified RNAs, stabilizing NANOG expression [62]. Collectively, modulation of FTO, ALKBH5, or their downstream targets restores drug sensitivity and limits tumor relapse in preclinical models. FTO has also been implicated in leukemic CSCs survival and resistance to differentiation therapies [104]. By demethylating transcripts such as ASB2 and RARA, FTO promotes survival and resistance to differentiation therapies in acute myeloid leukemia [104]. ALKBH5 supports the maintenance of glioblastoma cancer stem-like cells by stabilizing FOXM1 transcripts [103]. In breast cancer, ALKBH5 has been shown to enhance NANOG expression, contributing to hypoxia-induced CSC enrichment [105]. All these findings illustrate how “erasers” allow CSCs to adapt to tumor microenvironmental cues, such as low oxygen levels or chemotherapeutic stress and the therapeutic potential of targeting epitranscriptomic modifications.

### 3.3. “Readers”

Finally, the functional impact of m^6^A is determined by “readers”, proteins that recognize methylated transcripts and dictate their fate. The YTH (YT521-B homology) domain family proteins (YTHDF1/2/3 and YTHDC1/2) are the best characterized, with roles in translation promotion, mRNA decay and nuclear processing, shaping the balance between differentiation and self-renewal [106]. Another important reader family, the Insulin-like growth factor 2 mRNA-binding proteins (IGF2BP1–3), stabilizes methylated mRNAs and enhances their translation, frequently promoting oncogenic programs [80]. Among these m^6^A “readers”, YTHDF1/2 and IGF2BP2/3 regulate mRNA stabilization and translation of stemness-related genes in gastric cancer. YTHDF2 destabilizes ONECUT2 mRNA, indirectly activating TFPI and promoting stemness and oxaliplatin resistance [70]. IGF2BP2 stabilizes colony stimulating factor 2 (CSF2) mRNA, driving MSC reprogramming and enhancing stemness, proliferation, and invasion via Notch suppression [86]. HNRNPA2B1 (Heterogeneous nuclear ribonucleoproteins A2/B1) has emerged as another critical reader, promoting metabolic rewiring and therapy resistance [93]; its knockdown reduces tumorsphere formation, stemness marker expression, and increases response to cisplatin [94]. Mechanistically, hnRNPA2B1 regulates BIRC5 splicing, stabilizes NEAT1, and activates Wnt/β-catenin signaling, thereby sustaining CSC traits and chemoresistance [95]. Collectively, these m^6^A “readers” act as post-transcriptional regulators that reinforce oncogenic programs driving CSC identity. In hepatocellular carcinoma, IGF2BP1 stabilizes c-MYC mRNA in an m^6^A-dependent manner, sustaining cancer stem-like features [80]. IGF2BPs also contribute to chemoresistance and metastasis in multiple cancers, underscoring their role as oncogenic amplifiers [107]. Through all these “readers”, CSCs exploit m^6^A marks to amplify pathways such as WNT, NOTCH, or MYC signaling, sustaining self-renewal and therapy resistance.

### 3.4. Clinical Relevance of the m^6^A Molecular Pathway

Evidence across tumor types highlights the versatility of the m^6^A pathway in CSC biology and shows that CSCs rely on the flexibility of its components to maintain their identity and adapt under selective pressures. The clinical relevance of this pathway is increasingly evident, as aberrant expression of m^6^A regulators such as METTL3, FTO, ALKBH5, or IGF2BPs correlates with poor prognosis, advanced disease stage, therapy resistance and immune evasion, in multiple cancer types [108,109,110,111]. In gastric cancer, global dysregulation of the m^6^A machinery has also been documented, with METTL3, FTO, YTHDFs, IGF2BPs and hnRNPA2B1 frequently upregulated in patient tissues and associated with poor prognosis, advanced stages and metastasis [42]. Conversely, downregulation of METTL14 expression correlates with unfavorable outcomes [42].

Beyond prognostic implications, the m^6^A machinery presents promising therapeutic opportunities. Strategies aimed at selectively targeting “writer”’s activity, disrupting “readers” or interfering with their RNA interactions are under active investigation [112,113,114,115,116]. By inhibiting METTL3, m^6^A marks on oncogenic mRNAs are reduced, destabilizing them and suppressing gastric cancer growth [117]. The METTL3 small molecule inhibitor STM2457 was firstly studied as a therapeutic approach in myeloid leukemia [118]. Importantly, STM2457 shows antitumor activity in preclinical models of gastric cancer, when combined with anti–PD-1 therapy [119]. Small-molecule inhibitors of FTO have also shown promise in gastric cancer, allowing the increase in m^6^A levels and regulating Wnt/PI3K-Akt signaling [120]. One of these inhibitors, meclofenamic acid, is already being used in a clinical trial enrolling patients with recurrent or progressive brain metastasis from solid primary tumors (NCT02429570) and in preclinical leukemia models [121].

The m^6^A RNA modifications and their regulators—including METTL3, ALKBH5, FTO, YTHDFs, hnRNPA2B1, and IGF2BP2—have particularly emerged as key drivers of chemotherapy resistance in gastric cancer [86,94,122]. For instance, METTL3 “writer” is upregulated in oxaliplatin-resistant gastric cancer cells and promotes resistance via the DNA repair pathway [44,47]. The “eraser” ALKBH5 has been associated with chemotherapy resistance and stemness maintenance, suggesting that its expression levels might predict therapeutic response [94]. Equally important are the m^6^A “readers”, including YTHDF1-3, IGF2BP1-3 and hnRNPA2B1 proteins, which bind methylated transcripts to regulate their stability or translation to confer chemoresistance [94,95]. Elevated IGF2BP expression has been generally observed in gastric cancer and shown to stabilize oncogenic mRNAs, reinforcing stem-like traits [86]. These insights suggest that combining m^6^A modulators with chemotherapy may enhance therapeutic efficacy.

Besides driving CSC maintenance and therapeutic resistance, imbalanced m^6^A regulators promote immune evasion by modulating cytokine signaling, antigen presentation, and immune checkpoint expression, fostering an immunosuppressive tumor microenvironment. High expression of METTL3, METTL14, FTO, YTDHF1-2 and IGF2BP1, in gastric cancer, facilitates tumor immune evasion by maintaining the stability and expression of PD-L1 transcripts [123,124,125,126,127]. Increased activity of the IGF2BP1 “reader”, for instance, amplifies tumor proliferation and dampens CD8^+^ T-cell–mediated cytotoxicity, correlating with unfavorable patient outcomes. In contrast, its downregulation disrupts PD-L1–dependent immune suppression, thereby restoring antitumor immunity [127]. Loss of other m^6^A readers, such as YTHDF1, further influence immune modulation by enhancing dendritic cell recruitment and antigen presentation [128,129]. Demethylases such as ALKBH5 and FTO also reshape cytokine networks in gastric cancer [130]. ALKBH5 has been shown to modulate immune evasion by regulating CD8^+^ T-cell infiltration, CD58 expression, dendritic cell recruitment, and broader immune cell interactions [131,132]. FTO instead regulates TGF-β expression, but also correlates with poor immune infiltration [133]. Evidence shows dynamic interactions between CSCs and immune cells in the tumor microenvironment, with certain immune cells promoting CSC expansion while enabling immune evasion [134].

**Figure 2:**
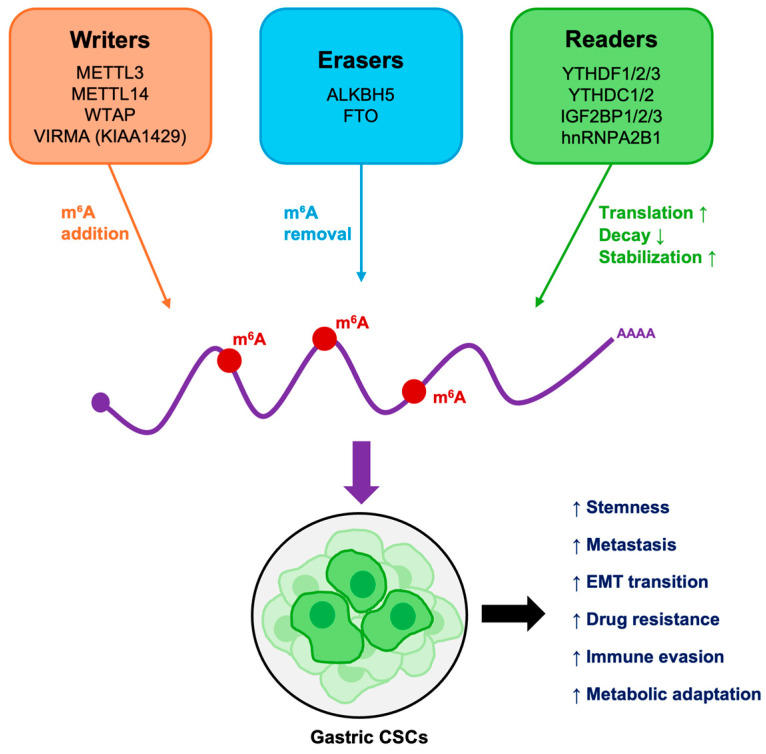
Functional overview of m^6^A-mediated regulation in GCSCs. m^6^A writers, erasers, and readers cooperatively regulate mRNA stability, translation, and degradation. Their dysregulation sustains stemness, promotes epithelial–mesenchymal transition (EMT), alters metabolism and immune responses, and drives therapy resistance and metastasis.

Collectively, these findings underscore the m^6^A-CSC axis as a pivotal regulator linking stemness, therapeutic resistance and immune escape (Figure 2). The m^6^A modification machinery provides CSCs with a powerful mechanism to control RNA fate and thereby sustain stemness, plasticity, and survival. While different cancers exploit different nodes of this machinery, the principle remains consistent: the balance between “writers”, “erasers”, and “readers” dictates CSC function and tumor behavior. As our understanding advances, targeting the m^6^A RNA methylation pathway, through pharmacological inhibition or silencing of its machinery, has emerged as a potential strategy to eliminate CSCs, overcome therapy resistance and enhance responses to chemotherapy and immunotherapy, offering new avenues for precision treatment also in gastric cancer [110,123,125].

## 4. Functional Role of m^6^A in Gastric Cancer Stem Cells

Functionally, METTL3, one of the best characterized m^6^A “writers”, appears to contribute to gastric cancer progression by stabilizing oncogenic transcripts and modulating key signaling pathways. Reported mechanisms include: (i) stabilization of HDGF mRNA via IGF2BP3, which enhances glycolysis and angiogenesis [45]; (ii) methylation of ZMYM1, which contributes to the inactivation of the RAS/ERK/c-FOS pathway and reduces E-cadherin expression, thereby facilitating EMT and metastasis [135]; (iii) promotion of ADAMTS9 degradation via YTHDF2, resulting in PI3K/AKT pathway activation and enhanced tumor progression [136]. Collectively, these processes may reinforce of a GCSC phenotype, characterized by increased therapy resistance, invasiveness and metastatic potential. Overall, current evidence suggests that m^6^A modifications in GCSCs function across multiple regulatory layers, from the fine-tuned editing of non-coding RNAs to the broader remodeling of oncogenic signaling networks [137]. These findings underscore that GCSCs are particularly reliant on m^6^A-driven transcriptional and post-transcriptional programs, which sustain their stem-like properties and underlie relapse and metastasis [17,41]. This dependency makes m^6^A regulators potential vulnerabilities of CSCs. Yet, because m^6^A also plays essential roles in normal physiology, therapeutic strategies must carefully exploit CSC-specific epitranscriptomic dependencies to achieve selectivity [138,139].

Additionally, several studies have also highlighted the diagnostic potential of m^6^A regulators. Unlike CSCs conventional markers such as CD44, CD133 and EpCAM, which often lack specificity and can be expressed in normal progenitor cells, m^6^A-associated signatures may offer a more dynamic and mechanistic readout of tumor aggressiveness [140,141]. For instance, the role of METTL3 in stabilizing oncogenic transcripts such as HDGF and long non-coding RNAs (e.g., PSMA3-AS1, MIR22HG) is strongly linked to GCSC self-renewal and tumor initiation capacity [41,45]. Measuring METTL3 expression or its downstream m^6^A-dependent RNA modifications could thus serve as a biomarker for the presence of GCSC populations. Their diagnostic relevance lies not only in tissue expression patterns but also in their potential detection in liquid biopsies (e.g., circulating tumor cells, exosomal RNA), making them attractive candidates for non-invasive cancer monitoring [142,143]. What makes m^6^A particularly compelling in diagnostics is its contextual plasticity. Unlike static genetic mutations, RNA modifications are reversible and dynamically reflect cellular states, including stress, hypoxia, and therapy exposure. This means that an m^6^A-based diagnostic approach could capture the evolving nature of GCSCs during disease progression and treatment, enabling more precise patient stratification [144]. Recent advances in m^6^A profiling technologies, such as MeRIP-seq, m^6^A-CLIP, and nanopore direct RNA sequencing, are accelerating the identification of GCSC-specific methylation signatures (Table 2) [145]. These methods have enabled the identification, quantification and functional characterization of m^6^A marks across the transcriptome with increasing accuracy and depth.

Early detection methods, such as MeRIP-seq and m^6^A-seq, relied on antibody-based RNA immunoprecipitation, followed by next-generation sequencing, providing the first transcriptome-wide m^6^A maps in mammalian cells [145,149]. In gastric cancer, MeRIP-seq has been instrumental in revealing that METTL3- and METTL14-mediated m^6^A methylation regulates oncogenic lncRNA, such as PSMA3-AS1 and MIR22HG, whose site-specific m^6^A modifications enhance transcript stability and promote proliferation and stemness while suppressing apoptosis in GCSCs but were limited in resolution (~100–200 nt) [41,158]. Subsequent advances, including miCLIP and m^6^A-CLIP, achieved single-nucleotide precision, enabling a more accurate characterization of m^6^A topology in cancer-related transcripts [146,159]. More recently, nanopore-based direct RNA sequencing and targeted assays such as SELECT and SCARLET have improved the sensitivity and clinical applicability of m^6^A detection. Notably, these techniques have validated lncRNA methylation sites that are associated with poor prognosis and chemoresistance in gastric cancer patients [41]. Integrated with computational modeling, single-cell, and spatial transcriptomic analyses, these approaches are now enlightening how m^6^A dynamics influence intratumoral heterogeneity, cancer stem cell maintenance, and therapy response, marking a significant step towards translational and precision applications in gastric cancer [159,160,161]. These approaches may eventually enable clinicians to better distinguish aggressive, stem-like gastric cancers from less malignant subtypes, potentially complementing or even enhancing current molecular diagnostic panels [138,139,146]. Thereby, the same m^6^A signatures that maintain GCSC plasticity might also serve as dynamic biomarkers, capturing the evolving states of stemness, drug resistance, and tumor aggressiveness [80]. By integrating epitranscriptomic readouts into diagnostic platforms, whether through tissue analysis or liquid biopsy approaches, it may become possible to identify high-risk patients earlier, monitor therapeutic responses in real time, and stratify patients more precisely than is achievable with conventional static markers [140,141,162]. In this way, the functional role of m^6^A in GCSCs extends beyond fundamental tumor biology, suggesting that epitranscriptomic markers may represent a promising bridge between mechanistic insight and clinical application.

Interestingly, similar m^6^A-dependent mechanisms have been reported in esophageal cancer, an anatomically and molecularly related malignancy [163]. METTL3 is frequently upregulated and promotes tumor initiation, proliferation, and metastasis through the AKT and EGR1/Snail pathways, while also enhancing glutamine metabolism [163,164,165,166]. Other “writers” (METTL16, WTAP, KIAA1429, RBM15) are likewise overexpressed, suggesting a broader activation of the m^6^A writing machinery, whereas METTL14 loss correlates with poor differentiation and aggressive behavior suggesting a tumor-suppressive role similar to that seen in gastric cancer [164,165]. Among “readers,” YTHDF1, YTHDF3, and hnRNPA2B1 facilitate metastasis, proliferation, and lipid metabolism, and “erasers” (ALKBH5, FTO) influence cell cycle control and therapy resistance [163,167,168]. This conserved epitranscriptomic machinery in gastric and esophageal cancer appears to support cancer stem-like properties and therapy resistance and may hold potential as a basis for developing improved diagnostic and therapeutic strategies in upper gastrointestinal malignancies.

## 5. Challenges and Future Directions

Although significant advances have been made in understanding the role of m^6^A modifications in GCSCs, several challenges remain before these findings can be effectively translated into clinical practice. One of the major limitations arises from the technical constraints of current profiling methods. Standard approaches such as MeRIP-seq and m^6^A-CLIP provide only population-level and low-resolution data, obscuring the heterogeneity of m^6^A signatures across individual CSCs [147,166]. Recently, single-cell m^6^A sequencing and direct RNA nanopore approaches have begun to address this issue, offering unprecedented resolution of isoform-specific and cell-specific methylation patterns; however, these technologies remain costly, technically demanding and are not yet standardized for clinical application [169,170,171]. Tools such as CRISPR/dCas13-based targeted m^6^A editing now make it possible to experimentally manipulate RNA methylation at single sites, offering unprecedented precision to dissect how these modifications influence CSC biology. Another important challenge concerns the pleiotropic nature of m^6^A regulators. Core enzymes such as METTL3, ALKBH5 and IGF2BP proteins play essential roles not only in cancer but also in normal tissue homeostasis, raising concerns about toxicity and selectivity when targeting these proteins therapeutically [10,167]. Preclinical studies, such as the development of the first-in-class METTL3 inhibitor STM2457 in leukemia, have demonstrated that pharmacological disruption of m^6^A machinery can impair cancer stemness [172]. Nevertheless, extending these approaches to solid tumors, including gastric cancer, will require strategies that exploit CSC-specific or context-dependent vulnerabilities, thereby minimizing adverse effects [118,139,173]. The dynamic and reversible nature of m^6^A modifications presents both opportunities and difficulties for their use as biomarkers. On one hand, their plasticity reflects the evolving states of cancer stemness, drug resistance and adaptation to microenvironmental stressors such as hypoxia. On the other hand, this variability complicates the establishment of stable diagnostic markers. Liquid biopsy approaches, including circulating RNA and exosomal RNA analysis, hold promise for tracking such changes in real time, but robust validation in large patient cohorts remains lacking [80,168]. Looking forward, the field is moving toward integrative and translational approaches. Multi-omics frameworks that combine epitranscriptomic signatures with genomic, epigenetic, proteomic, and metabolomic data may provide more robust predictive models of GCSC biology and disease progression [174]. Advances in artificial intelligence and machine learning are expected to accelerate this process by enabling the discovery of complex m^6^A-based patterns that predict relapse or therapeutic response [175,176,177]. At the same time, the translational gap must be bridged through prospective studies, standardized detection protocols and early-phase clinical trials designed to test m^6^A modulators in combination with established therapies. In summary, while challenges remain in terms of resolution, therapeutic selectivity, and biomarker validation, the study of m^6^A modifications in GCSCs represents a rapidly evolving frontier. With continued technological innovation and clinical validation, the epitranscriptomic regulation of GCSCs is poised to transition from a mechanistic insight into a clinically actionable paradigm for the diagnosis and treatment of gastric cancer.

## 6. Conclusions

Over time, CSCs have shifted from a theoretical concept to a clinically relevant driver of relapse and therapy resistance. In gastric cancer, they represent a crucial challenge, yet also an opportunity for targeted intervention. The discovery of m^6^A RNA modification as a master regulator of GCSC biology has opened new lines of research linking epitranscriptomics to cancer progression, diagnostics and therapy. Current evidence indicates that GCSCs are particularly dependent on m^6^A-driven programs controlling stemness, signaling and adaptation to stress, making m^6^A regulators compelling candidates for selective therapeutic targeting. At the same time, the widespread physiological roles of m^6^A highlight the need for precision strategies that exploit GCSC-specific dependencies while minimizing toxicity. Advances in single-cell sequencing, liquid biopsy technologies and selective small-molecule inhibitors are expected to accelerate the translation of these insights into the clinic. Taken together, integrating epitranscriptomic knowledge into CSCs biology offers a promising path towards more accurate diagnostics and effective therapies for gastric cancer.

## Figures and Tables

**Table 1:** The core components of the m^6^A modification machinery, categorized as “writers,” “erasers,” and “readers”, implicated in GCSC maintenance and therapy resistance, highlighting their potential as diagnostic biomarkers and therapeutic targets. Their canonical functions in RNA metabolism are outlined alongside their roles in GCSCs features.

Regulatory Role	Regulators	Function in RNA Metabolism	Representative Roles in GCSCs	Clinical Relevance in Gastric Cancer	References
**“Writers”** (Add m^6^A)	METTL3	Regulates splicing, export, translation efficiency, stability	Stabilizes oncogenic RNAs (MYC; PSMA3-AS1; MIR22HG; SNHG3; PARP1) to sustain stemness and therapy resistance (oxaliplatin)	Overexpression linked to poor prognosis; potential biomarker for aggressive gastric cancer and therapeutic target	[41,43,44,45,46,47]
	METTL14	Forms heterodimer with METTL3; supports transcript processing	Influences pluripotency and EMT; suppresses stemness via ATF5/WDR74/β-catenin	Reduced expression linked to poor prognosis; candidate for patient stratification	[48,49]
	WTAP	Regulatory subunit of the methyltransferase complex; Enhances efficiency of m^6^A deposition and RNA stability	Enhances chemotherapy resistance through TGF-β	Correlation with advanced stage and poor survival	[50,51,52,53,54]
**“Erasers”** (Remove m^6^A)	FTO	Modulates RNA stability, splicing and translation	Enhances stability of stemness-related RNAs (MYC and SOX2) and chemoresistance (5-FU)	Elevated expression predicts poor outcome; potential therapeutic marker and target	[55,56,57,58,59]
	ALKBH5	Regulates RNA stability, splicing, and translation	Enhances spheroid formation and upregulates stemness-associated markers (NANOG); drives cisplatin resistance	(Over/Under)-expression related to malignancy; considered, in some studies, a tumor suppressor	[60,61,62,63,64,65]
**“Readers”** (Bind and interpret m^6^A)	YTHDF1/2/3 YTHDC1/2	YTHDF1 increases RNA translation and stability; YTHDF2 promotes RNA decay; YTHDF3 facilitates RNA translation or degradation; YTHDC1 regulates splicing/export; YTHDC2 ensures RNA stabilization and translation	Regulate stemness and drug resistance (oxaliplatin, cisplatin and other platins; cyclophosphamide)	High expression consistently associated with worse survival, only YTHDF2 has a rather controverse role; related with antitumor immunity; emerging prognostic biomarkers and therapeutic targets	[44,66,67,68,69,70,71,72,73,74,75,76]
	IGF2BP1/2/3	RNA stability and translation; IGF2BP3 involved in regulating alternative splicing	Regulate self-renewal, expression of c-MYC, HMGA1 and resistance to therapies	IGF2BP1 can act as a tumor suppressor and IGF2BP2/3 as oncogenes, strongly correlated with metastasis and poor clinical outcome; prognostic markers	[45,77,78,79,80,81,82,83,84,85,86,87,88,89,90,91,92]
	hnRNPA2B1	Influences splicing and stability	Increase CSC traits, metabolic reprogramming, and chemoresistance by regulating BIRC5 and NEAT1/Wnt–β-catenin	Overexpression correlates with chemoresistance; candidate target for drug-resistant gastric cancer	[93,94,95]

**Table 2:** Summary of the main techniques used for m^6^A detection and their translational relevance.

Method	Principle	Clinical Relevance/Limitation	References
**MeRIP-seq/m^6^A-seq**	Immunoprecipitation of m^6^A-modified RNA fragments followed by sequencing	Identifies potential diagnostic or prognostic m^6^A signatures; limited by resolution and reproducibility for clinical validation	[146,147]
**MeRIP-qPCR/targeted RIP**	Immunoprecipitation of m^6^A followed by qPCR for selected transcripts	Adaptable for translational labs; feasible for small patient cohorts or validation of diagnostic markers	[148]
**miCLIP/m^6^A-CLIP**	UV crosslinking of anti-m^6^A antibody to modified bases for single-nucleotide mapping	Enables precise identification of clinically relevant modification sites; currently impractical for routine diagnostics	[149]
**Nanopore Direct RNA Sequencing (DRS)**	Long-read direct RNA sequencing detects modified bases through electrical signal shifts	Promising for non-invasive and real-time m^6^A biomarker detection in liquid biopsies; still early for clinical use	[149,150,151]
**LC–MS/MS (mass spectrometry)**	Quantitative detection of modified nucleosides in hydrolyzed RNA	Provides global m^6^A load; applicable to clinical QC and comparative studies but not to site-specific biomarker validation	[152]
**SCARLET/SELECT/site-specific PCR assays**	Enzymatic or ligation-based assays to quantify m^6^A at defined sites	Most suitable for clinical translation, enables targeted, reproducible quantification in tissues and liquid biopsies	[149,152,153]
**Single-cell/single-nucleus m^6^A profiling**	Adaptation of antibody- or enzyme-based m^6^A detection to single cells	Holds potential for identifying stem-like or drug-resistant tumor cells in mixed clinical samples	[154,155]
**Computational prediction and integration pipelines**	Motif-based modeling and integration with transcriptomic and clinical data	Supports biomarker discovery and prognostic model building from patient datasets (e.g., TCGA)	[156,157]

## Abbreviations

The following abbreviations are used in this manuscript:

ALKBH5	AlkB homolog 5
CSC	Cancer stem cell
EMT	Epithelial–mesenchymal transition
GCSC	Gastric cancer stem cell
FTO	Fat mass-and obesity-associated protein
hnRNPA2B1	Heterogeneous nuclear ribonucleoproteins A2/B1
IGF2BP1/2	Insulin-like growth factor 2 mRNA-binding proteins
lncRNA	Long non-coding RNAs
m^6^A	N^6^-methyladenosine
METTL3	Methyltransferase 3
METTL14	Methyltransferase 14
WTAP	Wilms tumor 1-associated protein
VIRMA	vir like m6A methyltransferase associated
YTHD	YT521-B homology domain
